# Adverse Childhood Experiences in Medical Students: Implications for Wellness

**DOI:** 10.1007/s40596-019-01047-5

**Published:** 2019-03-08

**Authors:** Andrés F. Sciolla, Michael S. Wilkes, Erin J. Griffin

**Affiliations:** University of California, Davis, Sacramento, CA USA

**Keywords:** Adverse childhood experiences, Burnout, Depression, Medical student resilience, Suicide

## Abstract

**Objective:**

The primary purpose of the study was to assess the prevalence of adverse childhood experiences (ACEs) in a cohort of third-year medical students and characterize their childhood protective factors.

**Methods:**

The authors developed a web-based anonymous survey distributed to all third-year medical students in one school (*N* = 98). The survey included the 10-item ACE Study questionnaire, a list of childhood protective factors (CPF) and questions to assess students’ perception of the impact of ACEs on their physical and mental health. The medical school’s IRB approved the student survey as an exempt study. The authors computed descriptive and comparative statistical analyses.

**Results:**

Eighty-six of 98 students responded (88% response rate). Forty-four students (51%) reported at least one ACE exposure and 10 (12%) reported ≥ 4 exposures. The latter were all female. The average difference in the ACE score between male and female medical students was − 1.1 (independent *t* test with unequal variances *t*(57.7) = − 2.82, *P* = .007). Students with an ACE score of ≥ 4 were significantly more likely to report a moderate or significant effect on their mental health, compared with students with scores ≤ 3 (chi-square test, *P* = < .0001). Most students reported high levels of CPF (median score = 13 of a maximum score = 14). ACEs and CPF were inversely associated (Pearson correlation = − 0.32, *P* = .003).

**Conclusions:**

A sizeable minority of medical students reported exposure to multiple ACEs. If replicated, findings suggest a significant vulnerability of these medical students to health risk behaviors and physical and mental health problems during training and future medical practice.

Disturbingly high levels of burnout (defined as emotional exhaustion, feelings of detachment, and low sense of personal accomplishment associated with work-related stress), depression, and suicidal ideation among medical trainees [1, 2] have prompted calls for action focusing on changing approaches to medical education, screening, and early intervention strategies [3–7]. While many medical students appear resilient, research has identified personal characteristics associated with increased risk of poor mental health, including female gender [8], ethnic minority [8], sexual minority [9], and coping style [10]. However, a personal history of childhood adversity is a risk factor that remains understudied.

Studies in the general population and patient samples show that the cumulative effect of adverse childhood experiences (ACEs) is a robust predictor of poor physical and mental health in adulthood, including depression [11, 12] and suicidality [13, 14]. ACE usually refers to child abuse and neglect as well as indices of household (e.g., domestic violence) and sometimes neighborhood dysfunction (e.g., crime). ACEs do not affect all persons equally, and individual resilience (i.e., the ability to bounce back from adversity) has been shown to buffer the impact of childhood adversity on adult depression [15]. To our knowledge, there are no reports of the prevalence of childhood adversities in medical students assessed with comprehensive measures, such as the ACE Study questionnaire, which includes maltreatment (i.e., abuse and neglect) and other adversities (e.g., witnessing violence in the household). Knowledge of prevalence may advance research on individual developmental risk and resilience factors for poor mental health in medical students and lead to novel approaches to primary and secondary prevention.

The purpose of this exploratory study is to assess the prevalence of ACEs in third-year medical students at one public medical school on the West Coast. We expected ACE exposure to be lower in this medical school cohort than in the general population and in patient samples. Many medical students are children of parents with high educational attainment and economic success [16], and socioeconomic status and risk of child maltreatment are inversely related [17].

## Methods

### Subjects

All third-year medical students (*N* = 98) from one medical school on the West Coast of the USA were eligible to participate. The Institutional Research Review Committee for the school classified the student survey as an exempt study.

### Design

Cross-sectional. We sent students a unique link to a confidential email survey (SurveyMonkey®) close to the end of the third year of medical school (2015 academic year). The survey remained open over a two-week period. No identifying information was retained in the survey or associated with responses. All participants were informed of the voluntary nature of their participation prior to completing the survey. All de-identified data were electronically stored on a password-protected computer, ensuring confidentiality and anonymity.

### Setting

The survey was administered as preparation to a half-day, case-based, small group learning activity featuring a standardized patient reporting a history of childhood trauma. The primary educational objective of the survey was to promote reflection among students of their vulnerability vis-à-vis that of the standardized patient, a single mother presenting to a pediatrician worrying that her ACE exposure could affect negatively her child. In addition, we used the students’ answers regarding a list of protective factors during childhood to stimulate discussion on the role of childhood resources conferring resilience that may offset the risk stemming from ACE exposure.

### Measurements

The authors of the ACE Study took items from several previously validated measures to assemble a questionnaire tapping into childhood maltreatment (abuse and neglect) and household dysfunction [18]. The questionnaire has been widely used and validated in several languages. The items have forced-choice yes/no answers assessing respondents’ experiences before age 18. Four items contain a single question (e.g., “Did a household member go to prison?”), and six items contain two or more questions linked together by the “or” conjunction. Each “yes” was given 1 point, so the ACE score ranges from 0 to 10. We added two questions aimed at eliciting the respondents’ perception of the overall impact of childhood exposures on their physical and mental health. The questions were “When considering the ‘yes’ [ACE] items collectively, how much of an effect do you think they have had on your overall *physical* health?” and “When considering the ‘yes’ [ACE] items collectively, how much of an effect do you think they have had on your overall *mental* health?” Respondents were asked to rank their responses using a Likert-type scale with four responses: no effect, minimal effect, moderate effect, and significant effect.

To identify childhood protective factors (CPF), we used an inventory developed in 2006 by Mark Rains and Kate McClinn, two psychologists working at the Southern Kennebec Healthy Start, Augusta, ME [19]. The inventory consists of 14 statements with answers in a Likert-type format. Examples of protective statements include “When I was a child, there were relatives in my family who made me feel better if I was sad or worried” and “We had rules in our house and were expected to keep them.” For each statement, respondents were asked to select one of five responses: definitely true, probably true, not sure, probably not true, and definitely not true. A composite score was calculated by assigning 1 point to each statement answered “definitively true” or “probably true” and 0 point to “not sure,” “probably not true,” and “definitively not true” responses. The CPF scores range from 0 to 14. Although the psychometric properties of this inventory have not been tested, it contains several factors that research has shown to be linked consistently to resilience [20], including a study in college students with a history of ACEs [21].

### Data Analysis

We conducted quantitative analyses to obtain basic descriptive statistics, *t* tests and chi-square tests using Fisher’s exact test (due to small cell sizes) to examine differences in ACE and CPF scores between men and women. We also obtained the Pearson correlation between total ACE scores and total CPF scores and plotted individual scores in a scatterplot to visually examine the distribution of the total ACE scores by total resilience scores. We divided the scatterplot into quadrants to better understand the distribution of ACE scores by CPF scores in light of the implications of high versus low ACE scores by high versus low CPF scores. The analysis for this paper was generated using SAS software (version 9.4, copyright © 2002–2012, SAS Institute Inc., Cary, NC, USA).

## Results

The response rate was 88% (86 of 98 students) of whom 44 (51%) were female. Six nonrespondents were on personal or academic leave at the time the survey was administered. Responses to the ACE Study questionnaire are listed in Table 1. Across all 10 items, 42 students (49%) had a total of zero; 17 (20%) had a score of one; 10 (12%) had a score of two; 7 (8%) had a score of three; and 10 (12%) had a score ≥ 4. Students with a score ≥ 4 were all women.

**Table 1:** Distribution of ACE scores and responses to ACE Study questionnaire items by gender. The average total number ACE items and the frequency distribution of the number of ACE items (number and percent) are shown by gender and overall in the upper portion of the table. The frequency distribution (number and percent) for each ACE item is shown by gender and overall in the lower portion of the table

	Male no. (%)	Female no. (%)	Total no. (%)
ACE score	Mean = .76	Mean = 1.86	Mean = 1.33*
0	23 (54.76)	19 (43.18)	42 (48.8)
1	9 (21.43)	8 (18.18)	17 (19.8)
2	7 (16.67)	3 (6.82)	10 (11.6)
3	3 (7.14)	4 (9.09)	7 (8.1)
4	0	4 (9.09)	4 (4.6)
5	0	1 (2.27)	1 (1.2)
6	0	0	3 (3.5)
7	0	3 (6.82)	0 (0)
8	0	1 (2.27)	1 (1.2)
9	0	1 (2.27)	1 (1.2)
10	0	0	0
ACE category			
Emotional abuse	4 (10)	14 (32)	18 (21)**
Physical abuse	2 (5)	9 (11)	11 (13)**
Sexual abuse	4 (10)	9 (20)	13 (15)
Emotional neglect	1 (2)	6 (14)	7(8)
Physical neglect	2 (5)	3 (7)	5 (6)
Mother treated violently	0 (0)	6 (14)	6 (7)**
Substance abuse in household	4 (10)	9 (21)	13 (15)
Mental illness in household	9 (21)	16 (36)	25 (29)
Household member going to prison	2 (5)	4 (9)	6 (7)
Parental separation or divorce	4 (10)	6 (14)	10 (12)

*t test of overall mean difference by gender. For men, *M* = .76 ACEs, SD = 0.98; for women, *M* = 1.86 ACEs, SD = 2.39; *t*(57.7) = − 2.82, *P* = .007

**P < .05 Fisher’s exact test by gender

The average difference in the ACE score between male and female medical students was − 1.1 (independent *t* test with unequal variances *t*(57.7) = − 2.82, *P* = .0068). Female students reported higher rates than male students for each ACE category, although only three reached statistical significance: emotional and physical abuse and witnessing their mother or stepmother being physically abused. No male medical students endorsed this ACE item while six (14%) female medical students did so.

With regard to the students’ perception of the impact of ACE exposure on their mental health, 100% of students with an ACE score of ≥ 4 (*n* = 10) reported a moderate or significant effect on their mental health as compared with 26% of students with an ACE score of ≤ 3 (chi-square test using Fisher’s exact test, *P* = < .0001). When students were asked if they perceived a moderate or significant impact of ACE exposure on their physical health, the difference between students with an ACE score of ≥ 4 (30%) and students with an ACE score of ≤ 3 (12%) was not statistically different.

The overwhelming majority of students endorsed the “definitely true” and “probably true” responses to each of the statements in the list of CPF (median score = 13 of a maximum score = 14). There was no statistically significant gender difference in an average number of CPF endorsed. The Pearson correlation between the total ACE scores and the total CPF scores was − 0.31740 (*P* = .003): ACE and CPF scores were inversely related—higher ACE scores were associated with a lower number of protective factors, − 0.32 (*P* = .003).

**Fig. 1:**
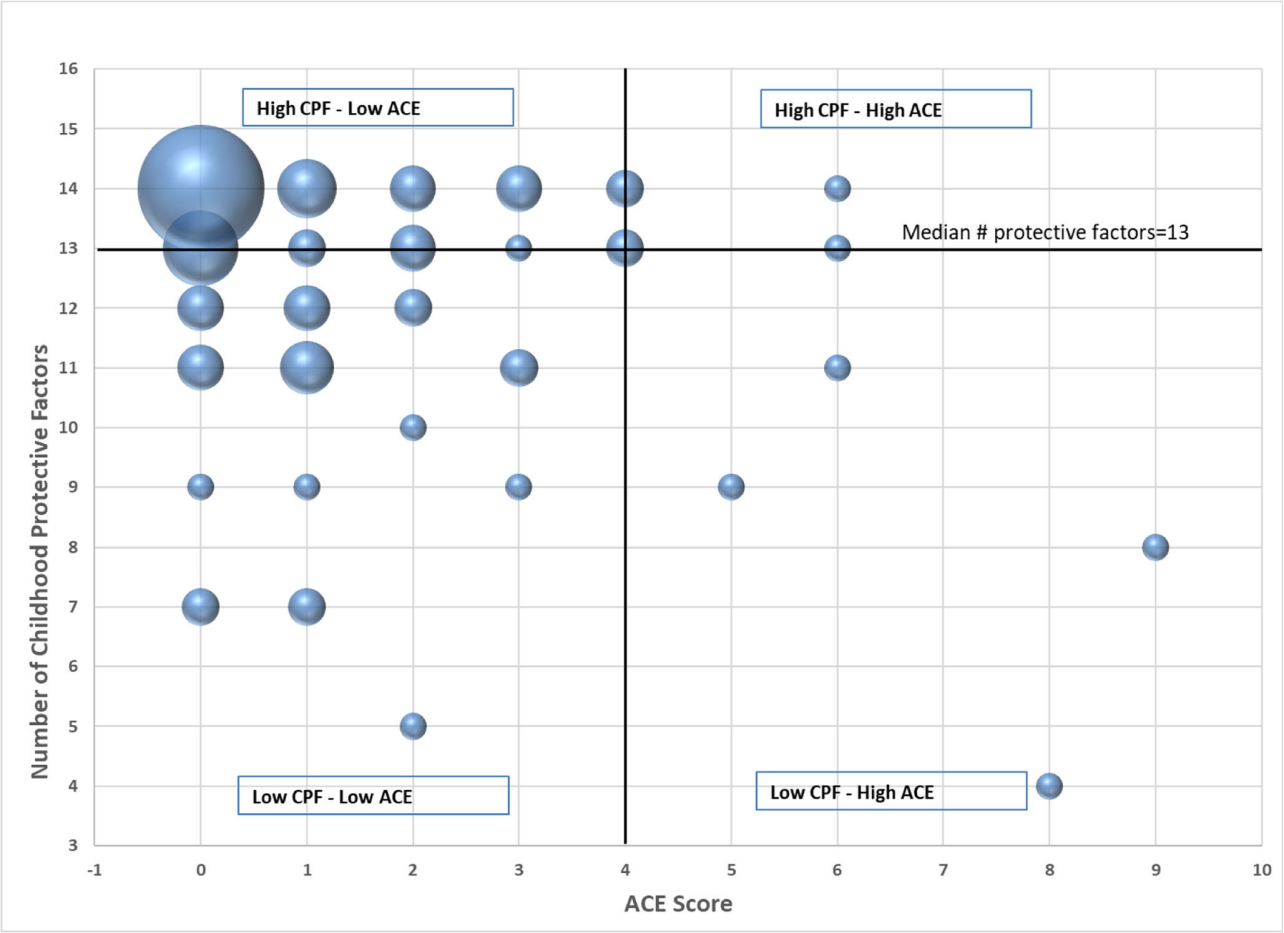
Bubble plot of ACEs and childhood protective factors for third-year medical students. The total number of ACEs is shown on the horizontal (*X*) axis, and the childhood protective factor score is shown on the vertical (*Y*) axis. The size of the *X*-*Y* data marker corresponds to the number of respondents with the corresponding ACE and protective factor score combination. Graphic was created in Microsoft Excel

When we plotted CPF endorsed as “definitely true” and “probably true” against ACE scores, data fell into four quadrants (Fig. 1). Most students fell within the top left quadrant—those with a high number of CPF and low ACE scores.

## Discussion

Our study from one medical school class suggests that one in five (20%) students have been exposed to three or more ACEs and one in 10 (12%) to more than four. These prevalence rates suggest an increased risk for burnout and depression on one hand and, on the other, problems in academic performance, as suggested by research in college students [22]. Disturbingly, students with an ACE score ≥ 4 were all women. However, almost one in four (23.8%) male medical students had an ACE score ≥ 2, and it is unknown whether a certain ACE score or exposure to a specific type of ACE increases significantly men’s risk to burnout. Moreover, nondisclosure of sexual abuse may be higher among male medical students as suggested by studies of adults in the general population [23]. Contrary to our expectations, the prevalence of ACEs in a class of third-year medical students was comparable to rates in the general population: in one study of five US states, 55.4% reported ≥ 1 ACEs and 13.7% reported ≥ 4 ACEs [24]. As a point of comparison, the corresponding rates in the landmark ACE Study conducted in a primary care setting were 52.1% and 6.2%, respectively [25].

Prior research suggests a high predictive value for health concerns when ACE scores are ≥ 4 [24, 25]. In our sample, 10 out of 86 students (12%) exhibited that level of risk. In the absence of data to the contrary, these findings suggest a need for concern about the vulnerability of these medical students with regard to health risk behaviors and physical and mental health problems during training and, in the future, in medical practice.

Gender differences in our sample were similar to those reported in the literature, with higher overall ACE scores among women compared with men in the general [24] and patient populations [25]. Examining averages, however, obscures the fact that female students endorsed more exposure than male students for each ACE category. Moreover, this difference reached statistical significance for three of the four ACE categories that entail abuse and violence (Table 1). Female physicians are twice more likely than male physicians to experience intimate partner violence [26], and research in college students shows that witnessing paternally perpetrated abuse is significantly related to intimate partner violence victimization for females, but not for male students [27]. Future studies should investigate whether female students whose mothers were treated violently during their childhood are at increased risk for intimate partner violence victimization.

Most students fell within the top left quadrant in Fig. 1, which depicts CPF endorsed as “definitely true” and “probably true” against ACE scores. We tentatively conclude that these are students at low risk for burnout and ill health. In contrast, we expect that students in the bottom right quadrant—those with high ACE scores and a low number of CPF—are at highest risk. We conclude that students with a high number of CPF and high ACE scores (top right quadrant) and those with a low number of CPF and low ACE scores (bottom left quadrant) are at intermediate risk. Although statistically significant and in the expected direction, the correlation coefficient of − 0.32 between CPF and ACE scores explains about 10% of variance. Our study is correlational and thus lacks statistical adjustment for additional risk and protective factors.

Resilience as a protective factor during medical school [28] and eventual practice [29] has received increased attention. Identifying trauma-informed approaches to build resilience by targeting psychological adaptations to ACEs is an area for future work [30]. Currently, there is evidence supporting the effectiveness of cognitive behavioral therapies, expressive writing, and mindfulness-based therapies for improving mental health and reducing health risk behaviors in adults with a history of ACEs, which can be delivered in resource-limited settings, such as primary care [31].

This exploratory study has important limitations. While the response rate was good, data collection occurred in only one class of students at one medical school and thus may not represent experiences of students in other classes or at other schools. The survey was administered in the second half of the third year of medical school—a period of noted academic intensity. However, questions focused on childhood experiences and responses were unlikely to be impacted by curricular workload or stress. Moreover, since the study lacked a comparison group, future studies would benefit from comparing medical students to students from other professions within health (e.g., nursing) or not (e.g., law). Because we were concerned about response burden in a pilot study, we used a broad question to assess the impact of ACE exposure on mental health. Future studies should employ valid measures of psychiatric assessment, either self-report or interviewer-based, to assess the impact of ACEs on medical students well-being. An additional limitation is the use of a measure of CPF whose psychometric properties have not been tested as a proxy for resilience. Future studies should use a validated measure of resilience. Although the assessment of childhood adversities retrospectively can be considered a limitation, the comparison of ACEs prospectively recorded throughout childhood and retrospectively recalled in adulthood shows moderate agreement, and, importantly, both assessment methods are correlated to objective and subjective measures of adult physical, mental, cognitive, and social health [32]. Lastly, we did not attempt to evaluate with validated measures the relationship between ACE scores and relevant outcomes, such as health risk behaviors, self-esteem, or academic performance, either currently or prospectively.

Even considering limitations, findings from this study point to the need for longitudinal research to follow students with elevated vulnerabilities to explore their health, academic outcomes, and postgraduation behaviors while characterizing protective factors with a validated measure of resilience. Given the great concern about medical trainees’ wellness, burnout, substance abuse, and even suicide, it seems essential to understand predictive and protective factors for those at greatest risk.

US medical schools are increasingly adopting admission practices with diversity and inclusion goals to ensure that future physicians are demographically and culturally well matched to the populations they serve. Perhaps, then, it is no surprise that the ACE scores of our increasingly diverse student body mirror those of the general population. Possibly, what is unique about these diverse students is their resilience. Now, it is up to medical school administrators and educators to match those strengths with the curricular interventions and healthcare resources that can help them address the vulnerabilities this study has begun to characterize.
